# Cantharidin Induced Oral Squamous Cell Carcinoma Cell Apoptosis via the JNK-Regulated Mitochondria and Endoplasmic Reticulum Stress-Related Signaling Pathways

**DOI:** 10.1371/journal.pone.0168095

**Published:** 2016-12-08

**Authors:** Chin-Chuan Su, Kuan-I Lee, Mu-Kuan Chen, Chun-Ying Kuo, Chih-Hsin Tang, Shing Hwa Liu

**Affiliations:** 1 Graduate Institute of Basic Medical Science, College of Medicine, China Medical University, Taichung, Taiwan; 2 Department of Otorhinolaryngology, Head and Neck Surgery, Changhua Christian Hospital, Changhua, Taiwan; 3 Department of Emergency, Taichung Tzu Chi Hospital, Buddhist Tzu Chi Medical Foundation, Taichung, Taiwan; 4 Institute of Toxicology, College of Medicine, National Taiwan University, Taipei, Taiwan; 5 Department of Medical Research, China Medical University Hospital, China Medical University, Taichung, Taiwan; Fu Jen Catholic University, TAIWAN

## Abstract

Oral cancer is a subtype of head and neck cancer which represents 2.65% of all human malignancies. Most of oral cancer is histopathologically diagnosed as oral squamous cell carcinoma (OSCC). OSCC is characterized by a high degree of local invasion and a high rate of metastasis to the cervical lymph nodes. How to prevention and treatment of OSCC is important and imperative. Here, we investigated the therapeutic effect and molecular mechanism of cantharidin, an active compound isolated from blister beetles, on OSCC *in vitro*. Results showed that cantharidin significantly decreased cell viability in human tongue squamous carcinoma-derived SAS, CAL-27, and SCC-4 cell lines. The further mechanistic studies were carried out in SAS cells. Cantharidin also significantly increased apoptosis-related signals, including caspase-9, caspase-7 and caspase-3 proteins. Besides, cantharidin decreased mitochondrial transmembrane potential (MMP) and induced cytochrome c and apoptosis inducing factor (AIF) release. Cantharidin also increased Bax, Bid, and Bak protein expressions and decreased Bcl-2 protein expression. Cantharidin could also increase the endoplasmic reticulum (ER) stress signals, including the expressions of phosphorylated eIF-2α and CHOP, but not Grp78 and Grp94. Furthermore, cantharidin reduced pro-caspase-12 protein expression. In signals of mitogen-activated protein kinases, cantharidin increased the phosphorylation of JNK, but not ERK and p38. Transfection of shRNA-JNK to OSCC cells effectively reversed the cantharidin-induced cell apoptotic signals, including the mitochondrial and ER stress-related signaling molecules. Taken together, these findings suggest that cantharidin induces apoptosis in OSCC cells via the JNK-regulated mitochondria and ER stress-related signaling pathways.

## Introduction

Oral cancer is one of the ten most common malignant human cancers. Although the etiology of oral squamous cell carcinoma (OSCC) is not fully understood, the risk factors for carcinogenesis are known to include tobacco use, alcohol, and betel quid chewing [1–4]. It has been estimated that every year approximately 263,000 new cases of OSCC occur worldwide and 127,000 people die from oral cancer [1, 5]. Over 90% of oral cancer is diagnosed as squamous cell carcinoma [3, 6]. Development of OSCC has a high potential for rapid and unlimited growth into tumor cells, and correlates with lymph node metastasis and poor 5-year survival rates [1, 7, 8]. It has been reported that the estimated new cases and deaths from lip and oral cavity cancers occurred in 2012 worldwide are 300,400 and 145,400, respectively; the age-standardized oral cavity cancer incidence rates per 100,000 in South-Eastern Asia are 4.0 in males and 2.5 in females [9]. In Taiwan, it has been estimated that the age-standardized incidence rate of OSCC is 146.2 per 100,000 person-years for areca/betel quid chewers [10]. Radiotherapy and chemotherapy are the main methods to treat OSCC; however, the prognosis is still poor [2, 11]. Thus, many studies have explored new therapeutic reagents and possible molecular mechanisms to potentially improve prognosis and therapy for OSCC patients, increasing their life quality and survival rate [1, 12–14].

Cantharidin is a pure and active compound isolated from *Cantharis vesicatoria* (blister beetles). The formulation specification for dried and ground blister beetle patches has been recorded in German Pharmacopeias. *Cantharis* is widely used for treatment of skin diseases, arthritis, rheumatism, and neuralgic pain in both complementary and alternative medicine [15]. Using gas chromatography and mass spectrometry, a post-mortem study in a fatal case of cantharides poisoning showed that serum cantharidin levels was about 72.3 ng/mL and the blister beetle powder contained about 0.87% of cantharidin [16]. In Chinese traditional medicine, doses of *Cantharis* are carefully managed to a range of 0.015–0.03 g to avoid serious systemic poisonous effects [15]. Cantharidin has been shown to induce apoptosis in many types of human cancer cell lines, including colon cancer, bladder cancer, pancreatic cancer, multiple myeloma and lung cancer [17–22]. The mechanisms of anti-apoptotic pathways have been suggested to contribute to the cancer development and the resistance of anticancer drugs [23]. The previous studies have found that cantharidin can enhance the mitochondria or endoplasmic reticulum (ER) stress-related apoptotic signals in lung cancer cells, lymphomas cells, and bladder cancer cells [19, 22, 24]. Cantharidin has also been shown to induce the inhibitory effects on murine ascites reticulum cell sarcoma and ascites hepatoma [25]. A clinical trial reported that cantharidin sodium, a semi-synthetic derivative of cantharidin, and Shenmai injection combined with chemotherapy in postoperative breast cancer patients significantly reduced the incidence of side effects (eg. leukopenia and gastrointestinal reactions) [26]. Norcantharidin, a demethylated analogue of cantharidin, has been suggested to induce cell apoptosis in human oral cancer cells via a mitochondria-mediated pathway [27]. However, the researches of cantharidin on OSCC are relatively fewer. The detailed effect and molecular mechanism of cantharidin on OSCC cell apoptosis still remain to be clarified. Based on findings from these previous studies, we hypothesized the potential for applying cantharidin to the treatment of OSCC. Cantharidin may induce apoptosis in OSCC cells through the mitochondria or ER stress-related signaling pathways. Therefore, in this study, we investigated the therapeutic effect and molecular mechanism of cantharidin on OSCC *in vitro*. Our results demonstrated that cantharidin induced both mitochondria and ER stress-related apoptotic signals in OSCC cells. Moreover, cantharidin-induced apoptosis was regulated by the mitogen-activated protein kinases (MAPK)/c-Jun NH2-terminal kinase (JNK) signaling. Cantharidin may be a useful chemotherapeutic reagent for the treatment of OSCC.

## Materials and Methods

### Chemicals, reagents and antibodies

Cantharidin (chemically pure compound), 3-(4,5-dimethyl thiazol-2-yl-)-2,5-diphenyl tetrazolium bromide (MTT), and dimethyl sulfoxide (DMSO) was purchased from Sigma-Aldrich (St. Louis, MO, USA). The antibodies used in the this study were directed against the following: caspase-9, caspase-7, caspase-3, glucose-regulated protein (Grp)78, eIF-2α, phospho-eIF-2α, cytochrome c, AIF, Bax, Bid, Bak, Bcl-2, JNK, phospho-JNK, p38, phospho-p38, extracellular signal-regulated kinase (ERK), phospho-ERK (Santa Cruz Biotechnology, USA), caspase-12 (Becton Dickinson, San Jose, CA, USA), Grp94, and C/EBP homologous protein (CHOP) (Cell Signaling Technology, USA). The cell culture medium included Dulbecco's modified Eagle's medium with 45% Ham's F12 medium and 10% fetal calf serum, all purchased from Gibco/Invitrogen (Carlsbad, CA, USA). The chemiluminescence reagents were purchased from Amersham Biosciences, Sweden. All other chemical reagents which were no specified in this study were obtained from Sigma-Aldrich.

### Cell culture

Three kinds of human tongue squamous carcinoma derived cells were used, including SAS cell line (a gift from Ph.D. Tzong-Der Way, China Medical University, Taichung, Taiwan), SCC-4 cell line (CRL-1624; American Type Culture Collection (ATCC)), and CAL-27 cell line (CRL2095; ATCC). The SAS cell line has been authenticated by short tandem repeat (STR) DNA typing (Mission Biotech, Taipei, Taiwan). Cells were cultured in a humidified chamber with a 5% CO_2_-95% air mixture at 37°C. The SAS and SCC-4 cells maintained in 45% Dulbecco's modified Eagle's medium with 45% Ham's F12 medium and 10% fetal calf serum. The CAL-27 cells were maintained in Dulbecco's modified Eagle's medium (DMEM) with 10% fetal calf serum (Gibco/Invitrogen, Carlsbad, CA, USA). In some experiments, the oral normal epithelium was obtained from non-tumor adjacent tissues of three OSCC male patients (41–48 years old) through debridement operation at Changhua Christian Hospital, Changhua, Taiwan. Human tissue sampling was approved by the Changhua Christian Hospital Institutional Review Board and written informed consent from the patients. The normal oral epithelial cells were isolated and cultured as described previously by Patil et al. [28]. The tissues were cut into smaller pieces and incubated with trypsin (0.025%) for 30 min, at 37°C. Cells were washed twice with phosphate-buffered saline (PBS) and centrifuged at 800 rpm for 5 min. Cells were maintained in DMEM containing 10% fetal bovine serum, which supplemented with epidermal growth factor 5 ng/mL, insulin 5 μg/mL, hydrocortisone 0.4 μg/mL, sodium selenite 5 ng/mL, and transferrin 10 μg/mL.

### Cytotoxicity assay

Cells were cultured in 24-well and incubated with various doses of cantharidin. After 24 hours, culture medium was removed and changed with fresh medium with 30 μL of 2 mg/mL MTT. After incubation for 4 h, the medium was removed, and 1 mL of DMSO was added to dissolve the blue formazan crystals. The viable cell number is directly proportional to the production of formazan. Following mixing, 150 μL was transferred to a 96-well plate. An enzyme-linked immunosorbent assay reader (Thermo Fisher Scientific, Waltham, MA, USA) was used for fluorescence detection at a wavelength of 570 nm [29].

### Genetic knockdown of JNK

The shRNA-JNK (5’-CAGTAAGGACTTACGTTGAAA-3’) in pLKO vector was purchased from National RNAi Core Facility Platform, Taipei, Taiwan. Cells were seeded into 6-well (5 x 10^5^/well) for overnight. The shRNA-control or shRNA-JNK (1 μg) was mixed with 1 mL of lipofectamine 2000. Cells were transfected with 2 μL of lipofectamine 2000 and shRNA-control or shRNA-JNK mixture for 24 h. Following transfection, cantharidin was added to fresh culture medium for further experiments.

### Western blot analysis

Cells were treated with cantharidin with or without shRNA transfection for various time periods. After, cells were lysed in RIPA-lysis buffer (Thermo Fisher Scientific) and cell lysates were collected. Fifty μg of protein from each cell lysate was subjected to electrophoresis on 10% (w/v) SDS-polyacrylamide gels and transferred to polyvinylidene difluoride membranes. The membranes were then blocked in PBST (PBS and 0.05% Tween 20) containing 5% nonfat dry milk for 1 h. For phosphorylated proteins, the membrane was blocked with 5% w/v BSA in TBST for 1 h. After blocking, the membrane was incubated with primary antibodies (1:1000), including procaspase-9, procaspase-7, procaspase-3, procaspase-12, cleaved caspase-3, cytochrome c, AIF, Bax, Bid, Bak, Bcl-2, phospho-eIF-2α, eIF-2α CHOP, Grp78, Grp94, phospho-JNK, JNK, phospho-ERK, ERK, phospho-p38, p38, α-tubulin. Membranes were then washed with 0.1% PBST and incubated with secondary antibodies conjugated to horseradish peroxidase for 45 min. The antibody-reactive bands were revealed using enhanced chemiluminescence reagents and exposed to radiographic film (Kodak, Rochester, NY, USA) [30, 31]. The protein expressions were quantified by densitometry and analyzed by ImageQant TL 7.0 software. (GE healthcare; Buckinghamshire, UK). The calculated protein expression data were a proportion of protein expression intensity to its internal control signal intensity. After, the protein fold of change could be compared in each experimental group.

### Mitochondrial transmembrane potential (MMP) assay

Cells were treated with or without cantharidin in the presence or absence of transfection of shRNA-control or shRNA-JNK. Cells were harvested and treated with DiOC_6_ (40 nM) for 30 min. The MMP was analyzed by using a FACScan flow cytometer (Becton Dickinson) to detect the DiOC_6_ fluorescence staining (Molecular Probes) [32].

We also used a cationic dye, tetraethylbenzimidazolylcarbocyanine iodide (JC-1) (Cayman), to detect MMP. Cells were cultured in 96-well (1 x 10^5^/well) and transfected with shRNA-control or shRNA-JNK for 48 h, and combined with or without cantharidin for 24 hours. Cells were washed twice and added 10 μL of JC-1 staining solution for 30 min, at 37°C. After plate was centrifuged for 400 x g, 5 min, the supernatant was aspirated and added 100 μL of assay buffer to each well. The ratio of fluorescence signals of healthy cells (excitation 535 nm/ emission 595 nm) to fluorescence signals of apoptotic cells (excitation 485 nm/emission 535 nm) was an indicator of apoptotic MMP depolarization. The fluorescence signal was detected by a fluorescence plate reader, SpectraMax^®^ (Molecular devices, CA, USA).

### Annexin-V-FITC assay

The cell apoptosis was determined by an annexin-V-FITC apoptosis detection kit (BioVision). Cells were cultured in 24-well plates (1 x 10^5^ cells/well) and treated with the indicated drugs for 24 h. The cells were washed twice with PBS and stained with annexin-V-FITC and propidium iodide (PI) for 20 min at room temperature. The fluorescence was detected by flow cytometry (FACScalibur, Becton Dickinson).

### Quantitative real-time polymerase chain reaction (qPCR) analysis

The qPCR experiments were performed as previously described [33]. For the detection of JNK-1 mRNA, qPCR was performed using real-time Sybr Green PCR reagent (Applied Biosystems, Foster City, CA, USA). Total cDNA (2 μL) was extracted from each sample and added to 25 μL reaction mixture containing sequence-specific primers and real-time Sybr Green PCR reagent. The human primers of JNK-1 are as follows, forward: (5'-3'): CTTGGCATGGGCTACAAGGA, and reverse: (5'-3'): TGGTGGAGCTTCTGCTTCAG (NCBI: NM_139049.2). The β-actin was used as a housekeeping gene for the internal control. The cycling conditions were as follows: 10 min of polymerase activation at 95°C, followed by 40 cycles of 15 s at 95°C and 60 s at 60°C. All amplification curves was analyzed with a normalized reporter (Rn: the ratio of the fluorescence emission intensity to the fluorescence signal of the passive reference dye) threshold of 0.2 to obtain the C_T_ (threshold cycle) values. The reference control genes were measured in quadruplicate in each PCR run, and their average C_T_ were used for relative quantification (the relative quantification method utilizing the real-time PCR efficiencies). The TF expression data was normalized by subtracting the mean C_T_ value of the reference gene from the C_T_ value of the test sample (ΔC_T_). The fold change was calculated using the formula 2^−ΔΔCT^, where ΔΔC_T_ represents ΔC_T-condition of interest_ − ΔC_T-control_. Prior to conducting statistical analyses, the fold change from the mean of the control group was calculated for each individual sample.

### Statistical analysis

The data are presented as the mean ± SEM. The statistical significance of the differences was evaluated by Student’s t-test. When more than one group was compared to one control, the significance was evaluated by one-way analysis of variance (ANOVA). The Duncan’s post hoc test was applied to identify group differences. Probability values below 0.05 were considered significant.

## Results

### Cantharidin reduces cell viability and promotes apoptotic signals in OSCC cells

**Fig 1:**
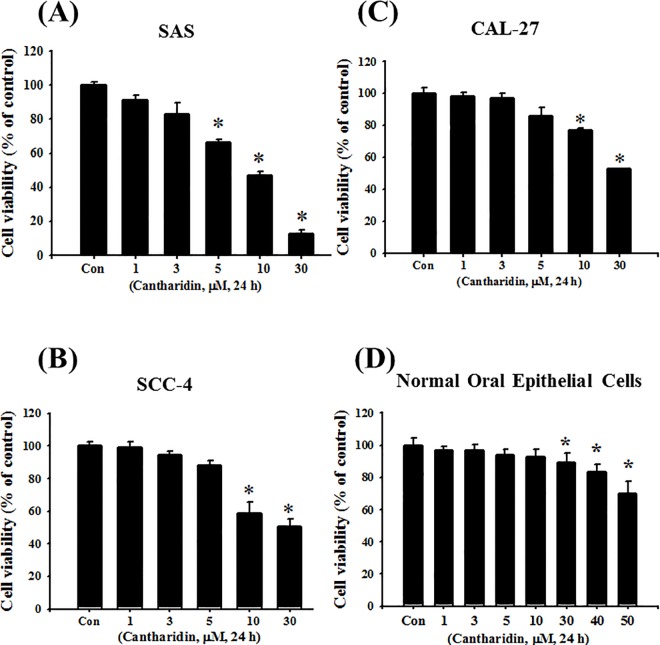
Effects of cantharidin on cell viability in SAS, CAL-27, and SCC-4 human tongue carcinoma cells and primary normal oral epithelial cells. Cells were treated with cantharidin (1 to 50 μM) for 24 h. The cell viability was subsequently analyzed by MTT assay. Data are presented as mean ± SEM of three independent experiments (n = 6). *P < 0.05 versus control group (Con).

**Fig 2:**
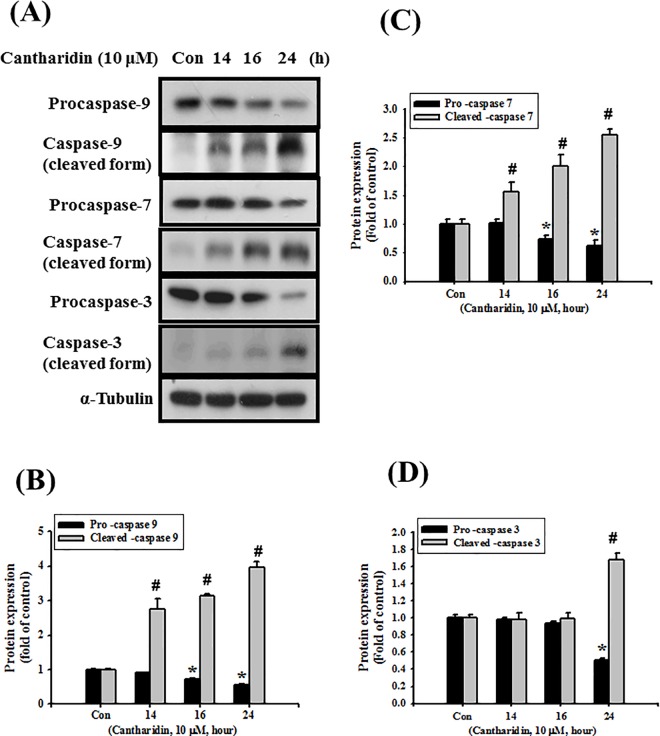
Effects of cantharidin on protein expressions of caspases in SAS human tongue carcinoma cells. Cells were treated with cantharidin (10 μM) for 14 to 24 h. (A) The protein expressions of pro-caspase-9, cleaved form of caspase-9, pro-caspase-7, cleaved form of caspase-7, pro-caspase-3, cleaved form of caspase-3 were determined by Western blotting. The protein expression of α-tubulin was as an internal control. In B-C, the protein expressions were quantified by densitometry and analyzed by ImageQant TL 7.0 software. Data are presented as mean ± SEM of three independent experiments (n = 6). *P < 0.05 versus control group for pro-caspases (Con). #*P* < 0.05 versus control group for cleaved form caspases.

To investigate the cytotoxicity effects of cantharidin on OSCC, cells (SAS, CAL-27, and SCC-4) were treated with 1 to 30 μM of cantharidin for 24 h and analyzed by MTT assay. The results showed that cantharidin significantly reduced SAS cell viability with the half maximal inhibitory concentration (IC50) of 10 μM (Fig 1A). The cantharidin could also induce cytotoxicity in SCC-4 and CAL-27 cells with the IC50 of about 30 μM (Fig 1B and 1C). Moreover, we also tested the cytotoxic effect of cantharidin on normal oral epithelial cells. As shown in Fig 1D, there was less cytotoxicity by cantharidin in normal oral epithelial cells than in OSCC cells. Next, we used the IC50 of cantharidin (10 μM) to detect apoptosis-related signals in SAS cells. Results showed that the protein expressions of procaspases 9, 7, and 3 were significantly reduced and the cleaved forms of caspases 9, 7, and 3 were significantly increased after cantharidin treatment for 14 to 24 h (Fig 2; *P* = 0.002 vs cleaved forms of caspases). These results indicated that cantharidin could activate the apoptotic pathway in OSCC cells.

### Cantharidin induces mitochondria- and ER stress-related apoptotic signals in SAS cells

**Fig 3:**
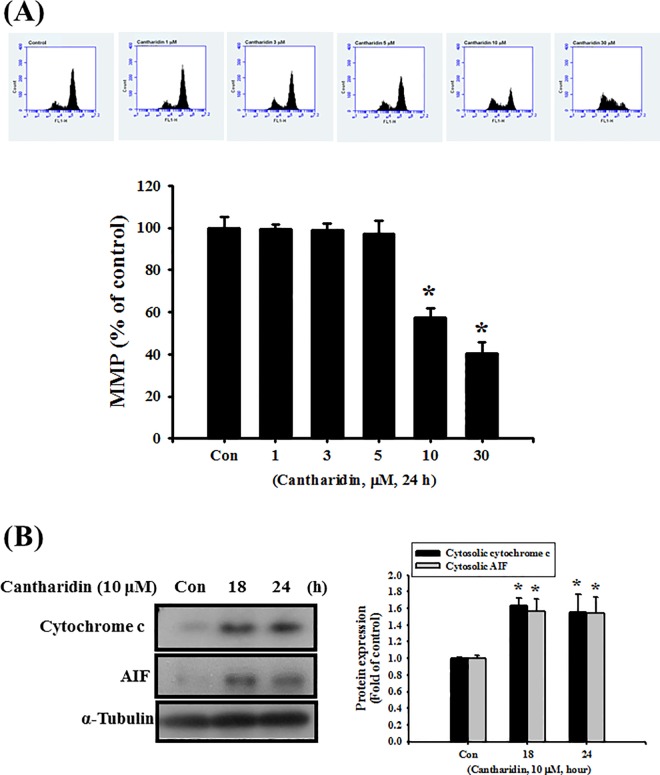
Effects of cantharidin on mitochondrial transmembrane potential (MMP) and protein expressions of cytochrome c and AIF in SAS human tongue carcinoma cells. (A) Cells were treated with cantharidin (1–30 μM) for 24 h. The MMP was analyzed by flow cytometry with a fluorescent dye DiOC_6_. (B) Cells were treated with cantharidin (10 μM) for 18 or 24 h. The cytosolic fraction was then subjected to Western blot analysis for cytochrome c and AIF. The protein expression of α-tubulin was as an internal control. The protein expressions were quantified by densitometry and analyzed by ImageQant TL 7.0 software. Data are presented as mean ± SEM of three independent experiments (n = 6). *P < 0.05 versus control group (Con).

**Fig 4:**
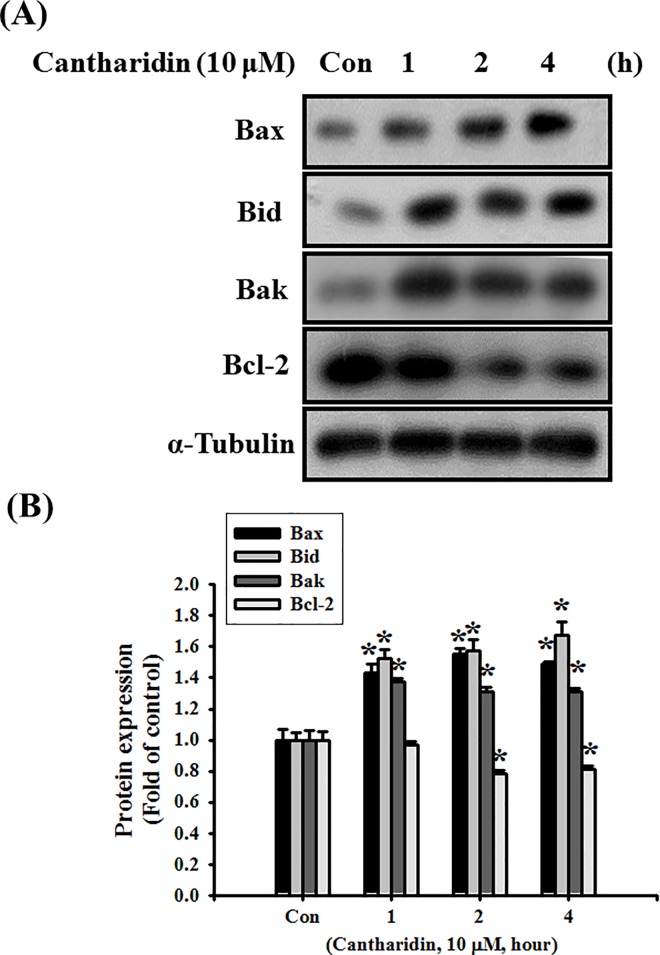
Effects of cantharidin on protein expressions of Bax, Bid, Bak, and Bcl-2 in SAS human tongue carcinoma cells. (A) Cells were treated with cantharidin (10 μM) for 1 to 4 h. The protein expressions were analyzed by Western blot analysis. The protein expression of α-tubulin was as an internal control. In B, the protein expressions were quantified by densitometry and analyzed by ImageQant TL 7.0 software. Data are presented as mean ± SEM of three independent experiments (n = 6). *P < 0.05 versus control group.

We next investigated whether cantharidin induced OSCC cell apoptosis through the mitochondria-related signaling pathways. As shown in Fig 3A, the MMP of SAS cells was significantly depolarized after treatment with 10 and 30 μM of cantharidin for 24 h (*P*<0.001 vs control). Moreover, both cytosolic cytochrome c and AIF release were also significantly increased following cantharidin administration (Fig 3B; *P*<0.001 vs control). The protein expressions of Bax, Bid, and Bak were significantly increased and the protein expression of Bcl-2 level was significantly decreased after administration of cantharidin (10 μM) for 1 to 4 h (Fig 4A and 4B; *P* = 0.002 vs control for Bax, Bid, and Bak; *P* = 0.03 vs control for Bcl-2).

**Fig 5:**
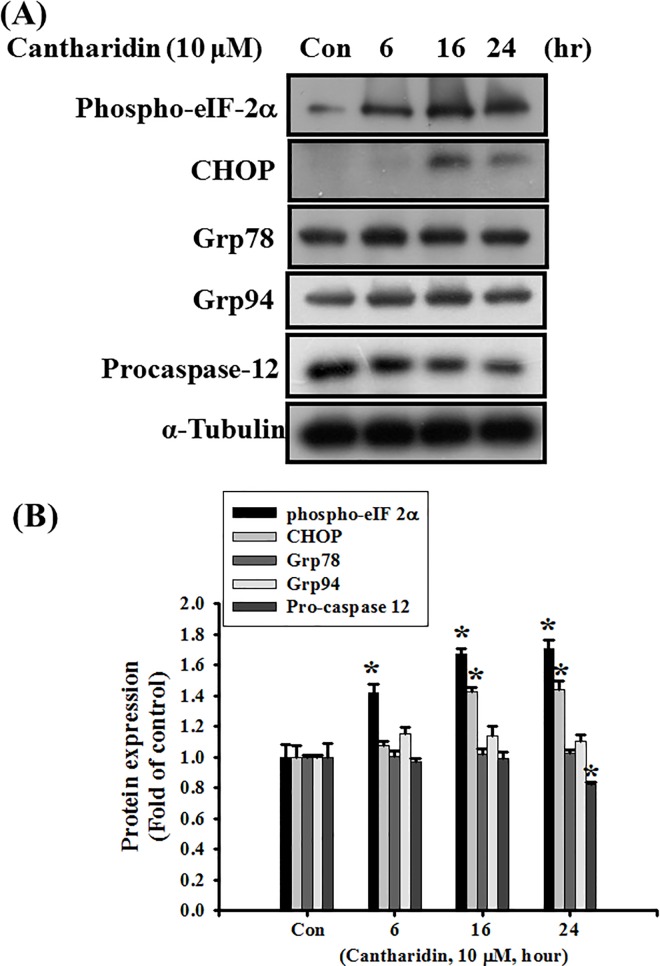
Effects of cantharidin on protein expressions of phospho-eIF-2α, CHOP, Grp78, Grp94, and procaspase-12 in SAS human tongue carcinoma cells. Cells were treated with cantharidin (10 μM) for 6 to 24 h. (A) The protein expressions of phospho-eIF-2α, CHOP, Grp78, Grp94 and procaspase-12 were analyzed by Western blotting. The protein expression of α-tubulin was as an internal control. In B, the protein expressions were quantified by densitometry and analyzed by ImageQant TL 7.0 software. Data are presented as mean ± SEM of three independent experiments (n = 6). *P < 0.05 versus control group.

The involvement of ER stress-related signals in cantharidin-induced OSCC cell apoptosis was further investigated. The results showed that cantharidin significantly increased the protein expressions of phospho-eIF-2α and CHOP and decreased the protein expression of procaspase-12, but did not alter the protein expressions of Grp78 and Grp94 (Fig 5; *P*<0.001 vs control for phospho-eIF-2α; *P* = 0.002 vs control for CHOP; *P* = 0.04 vs control for procaspase-12).

These results suggest that cantharidin may induce apoptosis in OSCC cells through the mitochondria- and ER stress-related signaling pathways.

### The involvement of JNK signaling in cantharidin-induced OSCC cell apoptosis

**Fig 6:**
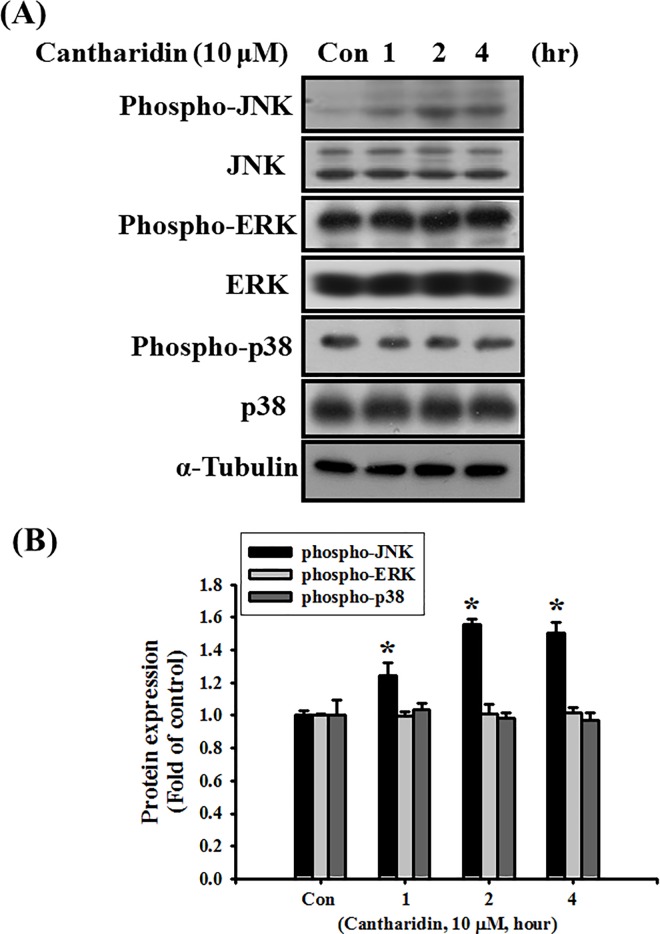
Effects of cantharidin on protein expressions of JNK, ERK, and p38 and their phosphorylation in SAS human tongue carcinoma cells. Cells were treated with cantharidin (10 μM) for 1 to 4 h. (A) The protein expression of JNK, ERK, and p38 and their phosphorylation were analyzed by Western blotting. In B, the protein expressions were quantified by densitometry and analyzed by ImageQant TL 7.0 software. Data are presented as mean ± SEM of three independent experiments (n = 6). *P < 0.05 versus control group for phospho-JNK.

To investigate the role of MAPK in the effects of cantharidin on SAS cell apoptosis, the phosphorylations of JNK, ERK, and p38 were determined. As shown in Fig 6, cantharidin significantly increased the phosphorylation of JNK in SAS cells, but did not affect the phosphorylations of ERK and p38 (*P*<0.001 vs control for phospho-JNK).

**Fig 7:**
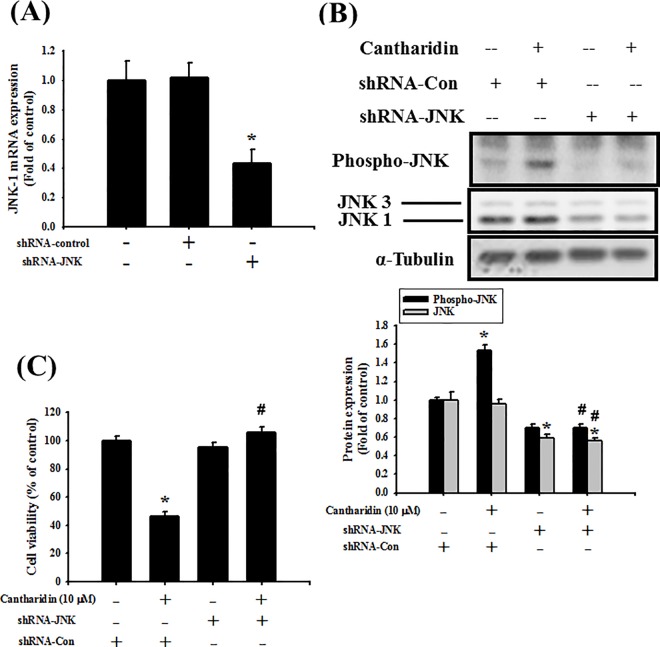
Transfection of shRNA-JNK inhibited cantharidin-induced JNK phosphorylation in SAS human tongue carcinoma cells. (A) Cells were transfected with sh-control (siRNA-con) or shRNA-JNK for 48 h, and the JNK-1 mRNA expression was detected by qPCR analysis. Data are presented as mean ± SEM of three independent experiments. *P < 0.05 versus sh-control group. (B) Cells were transfected with shRNA-JNK for 48 h, and then treated with cantharidin (10 μM) for 1 h. The JNK3/1 protein expression and phosphorylation were analyzed by Western blotting. The protein expression of α-tubulin was as an internal control. The protein expressions were quantified by densitometry and analyzed by ImageQant TL 7.0 software. Data are presented as mean ± SEM of three independent experiments. *P < 0.05 versus shRNA-control group. *P* < 0.05 versus shRNA-control with cantharidin group. (C) Cells were pretreatment with shRNA-JNK for 48 h, and then added cantharidin (10 μM) for 24 h. The cell viability was analyzed by MTT assay. Data are presented as mean ± SEM of three independent experiments (n = 6). *P < 0.05 versus sh-control group. #*P* < 0.05 versus shRNA-control combined with cantharidin group.

**Fig 8:**
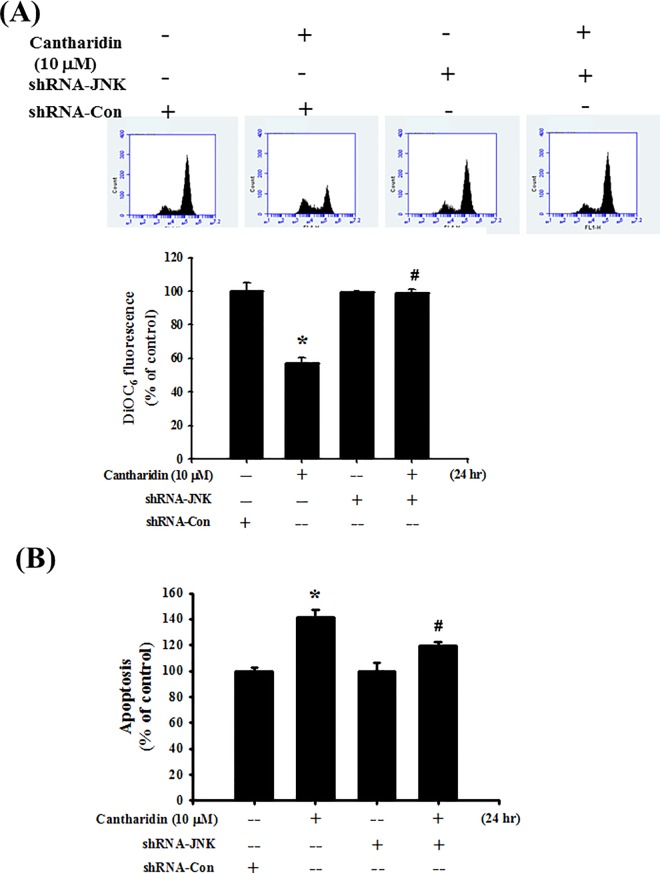
Transfection of shRNA-JNK inhibited cantharidin-induced MMP depolarization and apoptosis in SAS human tongue carcinoma cells. Cells were pretreatment with sh-control or shRNA-JNK for 48 h, and then added cantharidin (10 μM) for 24 h. Both MMP and apoptosis were analyzed by flow cytometry with the staining of DiOC_6_ and annexin V/PI, respectively. All data are presented as mean ± SEM of three independent experiments (n = 6). *P < 0.05 versus sh-control group. #*P* < 0.05 versus sh-control combined with cantharidin group.

**Fig 9:**
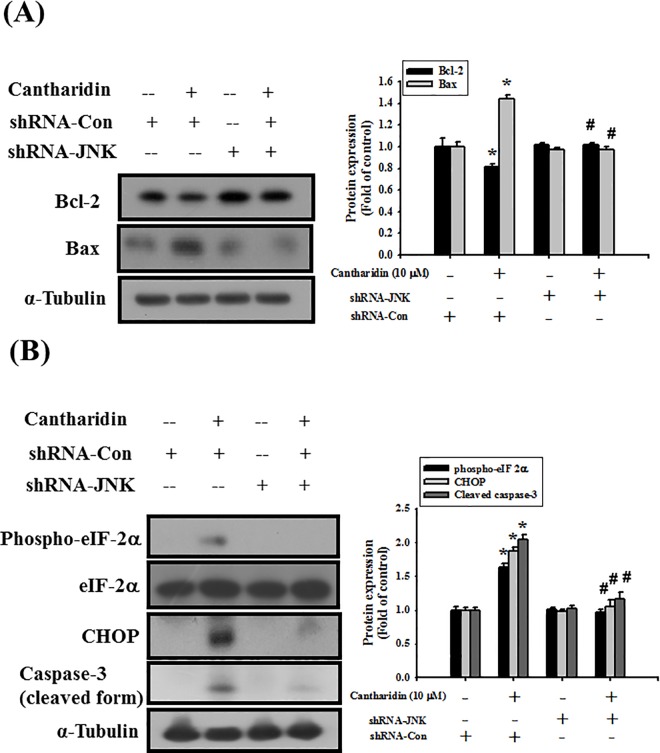
Transfection of shRNA-JNK reversed the effects of cantharidin on protein expressions of Bcl-2, Bax, phospho-eIF-2α, CHOP, and cleaved caspase-3 in SAS human tongue carcinoma cells. Cells were transfected with sh-control (siRNA-con) or shRNA-JNK for 48 h, and then treated with cantharidin (10 μM) for 4 h (A) or 24 h (B). The protein expressions of Bcl-2 and Bax (A) and phospho-eIF-2α, CHOP, and cleaved caspase-3 (B) were analyzed by Western blottingh. The protein expression of α-tubulin was as an internal control. The protein expressions were quantified by densitometry and analyzed by ImageQant TL 7.0 software. Data are presented as mean ± SEM of three independent experiments (n = 6). *P < 0.05 versus sh-control group. #*P* < 0.05 versus sh-control combined with cantharidin group.

To further investigate the role of JNK, the transfection of shRNA-JNK was used. In Fig 7A, cells transfected with shRNA-control did not affect JNK-1 mRNA expression, which was compared with control group. However, transfection with shRNA-JNK significantly decreased JNK-1 mRNA expression in SAS cells (Fig 7A; *P* = 0.002 vs shRNA-control). In next experiments, cells were transfected with shRNA-control or shRNA-JNK. The results showed that shRNA-JNK transfection significantly reversed the cantharidin decreased phosphorylation of JNK (Fig 7B; *P*<0.001 vs shRNA-control+cantharidin) and cells viability (Fig 7C; *P*<0.001 vs shRNA-control+cantharidin) in SAS cells. Further, the cantharidin-induced MMP depolarization (Fig 8A; *P*<0.001 vs shRNA-control+cantharidin) and apoptosis (Fig 8B and S1 Fig; *P* = 0.04 vs shRNA-control+cantharidin), decreased Bcl-2 protein expression, increased Bax protein expression (Fig 9A; *P*<0.001 vs shRNA-control+cantharidin), increased eIF-2α phosphorylation, increased CHOP protein expression, and increased caspase-3 cleavage (Fig 9B; *P*<0.001 vs shRNA-control+cantharidin) could also significantly reversed by the transfection of shRNA-JNK in SAS cells. We also used a cationic dye, JC-1, to confirm the effect of cantharidin on MMP. Cantharidin significantly induced MMP depolarization, which could be significantly reversed by the transfection of shRNA-JNK (S2 Fig).

## Discussion

OSCC is a common malignant cancer in the world. Although there are many clinical protocols for OSCC, such as surgical resection and radiotherapy, the overall 5-year survival rate remained poor [34]. A characteristic of mucosal lining cells is dividing rapidly and thus highly susceptible and damage to radiotherapy [35, 36]. Although patients received dental consultation prior to the initiation of radiotherapy, it still produced serious oral cavity problems that affect life quality [36]. Natural compounds have been preclinically and clinically investigated to be the chemopreventive or therapeutic agents for oral cancer [35, 37, 38]. Blister beetles are insects of family *Meloidae*, which contains about 2500 species, divided among 120 genera and 4 subfamilies (Eleticinae, Meloinae, Nemognathinae, and Tetraonycinae) [39, 40]. Cantharidin is a pure active compound, which is isolated from dried bodies of blister beetle. The concentration of cantharidin is various among species. The highest concentration of cantharidin is extracted from *Epicauta vittata*, which is 5.4% in dry weight bodies. While in the Spanish fly, the concentration of cantharidin almost reached to 5% in dry weight bodies [41]. Cantharidin has been reported to induce many poisoning symptoms during ingested, including burning of mouth, dysphagia, nausea, hematemesis, gross hematuria, dysuria, significant GI hemorrhage, renal dysfunction, coagulopathy, and cardiac dysrhythmias [40, 41]. However, cantharidin has been widely used in topical medication for warts, furuncles, and skin ulcer [15, 41]. Studies showed that cantharidin caused apoptosis in many kinds of cancer cells and possessed therapeutic potential in animal study and clinical trial for some cancers [25, 26, 42, 43]. Thus, cantharidin may be used to develop a preventive or therapeutic reagent for OSCC. However, the detailed effects and molecular mechanism of cantharidin on OSCC still remain to be clarified. In the present study, we demonstrated that cantharidin was capable of inducing OSCC cell apoptosis and death. Cantharidin induced OSCC cell apoptosis through the MAPK/JNK-regulated mitochondria and ER stress-related signaling pathways.

Apoptosis leads programmed cell death, which is a key regulator in physiological and homeostasis. Most of cancer therapies result in the activation of caspases. Activation of caspases initiates two pathways in cells. One is receptor pathway, which is the plasma membrane upon ligation of death receptor. Another is mitochondrial pathway [44–46]. Cantharidin derivative norcantharidin has been found to induce cytosolic cytochrome c accumulation and caspase-9 activation, but did not trigger Fas and FasL-related apoptosis [27]. In the present study, we found that cantharidin significantly decreased cell viability and induced apoptosis-related caspases activation in OSCC cells. The results for the increase of MMP depolarization, release of cytochrome c and AIF, Bcl-2 expression decrease, and Bax/Bid/Bak expressions demonstrated that cantharidin induced OSCC cell apoptosis through the mitochondria-dependent pathway.

Following chemicals or toxicants stimulation, it may change intracellular calcium homeostasis, protein synthesis, post-translational modification, and protein folding to lead ER stress. ER stress is occurred after excessive protein misfolding accumulation during protein biosynthesis [47]. After mitochondrial Bax and Bak protein expression, calcium is released from ER lumen into cytoplasm and subsequently induces calpain activation, triggering ER stress-related apoptosis [48–50]. In this study, we found that cantharidin significantly altered ER stress-related molecules expressions, including increased eIF-2α phosphorylation, increased CHOP protein expression, and decreased pro-caspase-12 protein expression, although the protein expressions of Grp78 and Grp94 were not changed. On the other hand, the initiation of eIF-2α phosphorylation is known to arrest protein translation, which reduces the loading into ER and restoration of ER homeostasis. However, prolong of ER stress leads caspase-12 activation and mediates cell apoptosis [47]. CHOP is a 29 KDa protein, which is composed of N-terminal transcriptional activation domain and C-terminal basic-leucine zipper (bZIP) domain. The JNK, p38, and ERK belong to the MAPK family. CHOP serves as a substrate for p38 MAPK. The activation of CHOP by p38 MAPK causes alteration in gene expressions that trigger cell apoptosis [51, 52]. The phosphorylation of JNK, p38, and ERK has been found to be involved in the natural compound gingerol-induced apoptosis in human colon cancer cells [53]. Blockage of JNK activation has been shown to reduce cell apoptosis in various cancer cells, such as prostate cancer cells, breast cancer cells, pancreas cancer cells, and colon cancer [53–57]. In the present study, we found that cantharidin significantly induced phosphorylation of JNK, but not ERK and p38 in OSCC cells. It seems that JNK activation may be involved in the cantharidin-induced cytotoxicity. Transfection of shRNA-JNK to block JNK expression and activation could significantly reverse the MMP depolarization, decreased Bcl-2 protein expression, increased Bax protein expression, eIF-2α phosphorylation, CHOP induction, caspase-3 cleavage induced by cantharidin in OSCC cells. These findings indicate that JNK signaling is involved in the cantharidin-induced OSCC cell apoptosis.

A fatal case of cantharides poisoning showed that the concentration of cantharidin in post-mortem serum was 72.3 ng/mL [16]. Nevertheless, two non-fatal cases of cantharidin poisoning has been reported that cantharidin induces acute toxicity at high concentrations (approximately 75 and 175 mg), producing nausea, severe lips, mouth, tongue, throat, and abdominal pain, dysuria, hematuria, renal tubular necrosis, hepatic degeneration, and myocarditis [58]. Cantharidin has been shown to induce cytotoxicity in carcinoma cells. The IC50 levels of cantharidin on human bladder carcinoma cell line T24 cells were 21.8, 11.2 and 4.6 μM for 6, 24 and 48 h, respectively [59]. Moreover, the IC50 level of cantharidin on tumor Hep 3B cells was 2.2 μM for 36 h; however, the IC50 level of cantharidin on normal Chang liver cells was 30 μM for 36 h [60]. It has been estimated that the selective index of cantharidin for normal Chang liver cells is 13 times higher than that for tumor Hep 3B cells [60]. An *in vivo* study showed that cantharidin (1.25–2 mg/kg, intraperitoneally or orally) possessed a definite inhibitory effect on murine ascites reticulum cell sarcoma and ascites hepatoma [25]. Moreover, several clinical trials have shown that cantharidin and its analogs combined with chemotherapy enhance clinical benefit response and reduce side effects of chemotherapy against breast cancer [26], gastric cancer [61], and primary hepatoma [62]. In the present study, cantharidin at the concentration of 10 μM effectively induced apoptosis and cell death in SAS OSCC cells via the JNK-regulated mitochondria and ER stress-related signaling pathways. Comprehensive understanding of the toxicological effects and mechanisms of cantharidin on tumor cells and normal cells may provide insight for the development of less toxic cantharidin analogues with good anti-cancer activity in the future.

**Fig 10:**
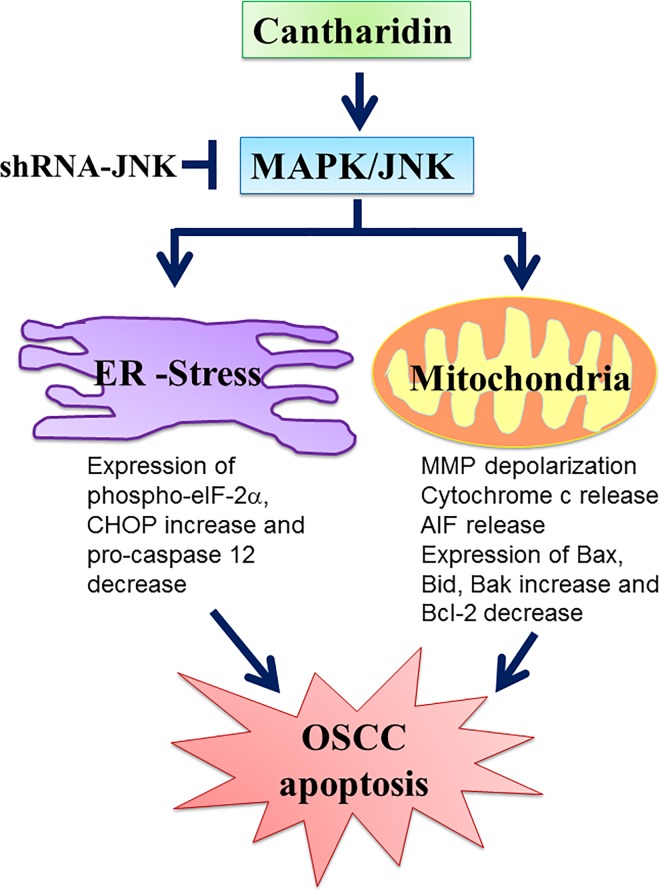
The schematic representation of proposed mechanisms of cantharidin on oral squamous cell carcinoma cells. Cantharidin induced cell apoptosis via the JNK-regulated mitochondrial and ER stress signaling pathways.

In conclusion, the current study presented the evidence showing that cantharidin decreased cell viability and activated apoptosis in OSCC cells via the mitochondria- and ER stress-related signaling pathways. This proposed molecular mechanism underlying the action of cantharidin is illustrated in Fig 10. These findings suggest that cantharidin possesses the possible chemotherapeutic potential for OSCC.

## Supporting Information

S1 Fig The 2D plots of annexin V and PI staining from flow cytometry for apoptosis in cantharidin-treated SAS human tongue carcinoma cells with or without shRNA-JNK transfection. (TIF)

S2 Fig Transfection of shRNA-JNK inhibited cantharidin-induced MMP depolarization in SAS human tongue carcinoma cells using a fluorescent JC-1 assay. (TIF)
