# Supplementary figures and images for: Cantharidin Induced Oral Squamous Cell Carcinoma Cell Apoptosis via the JNK-Regulated Mitochondria and Endoplasmic Reticulum Stress-Related Signaling Pathways
Source: PLoS One. 2016 Dec 8;11(12):e0168095. doi: 10.1371/journal.pone.0168095 (PMC5145211; doi:10.1371/journal.pone.0168095)

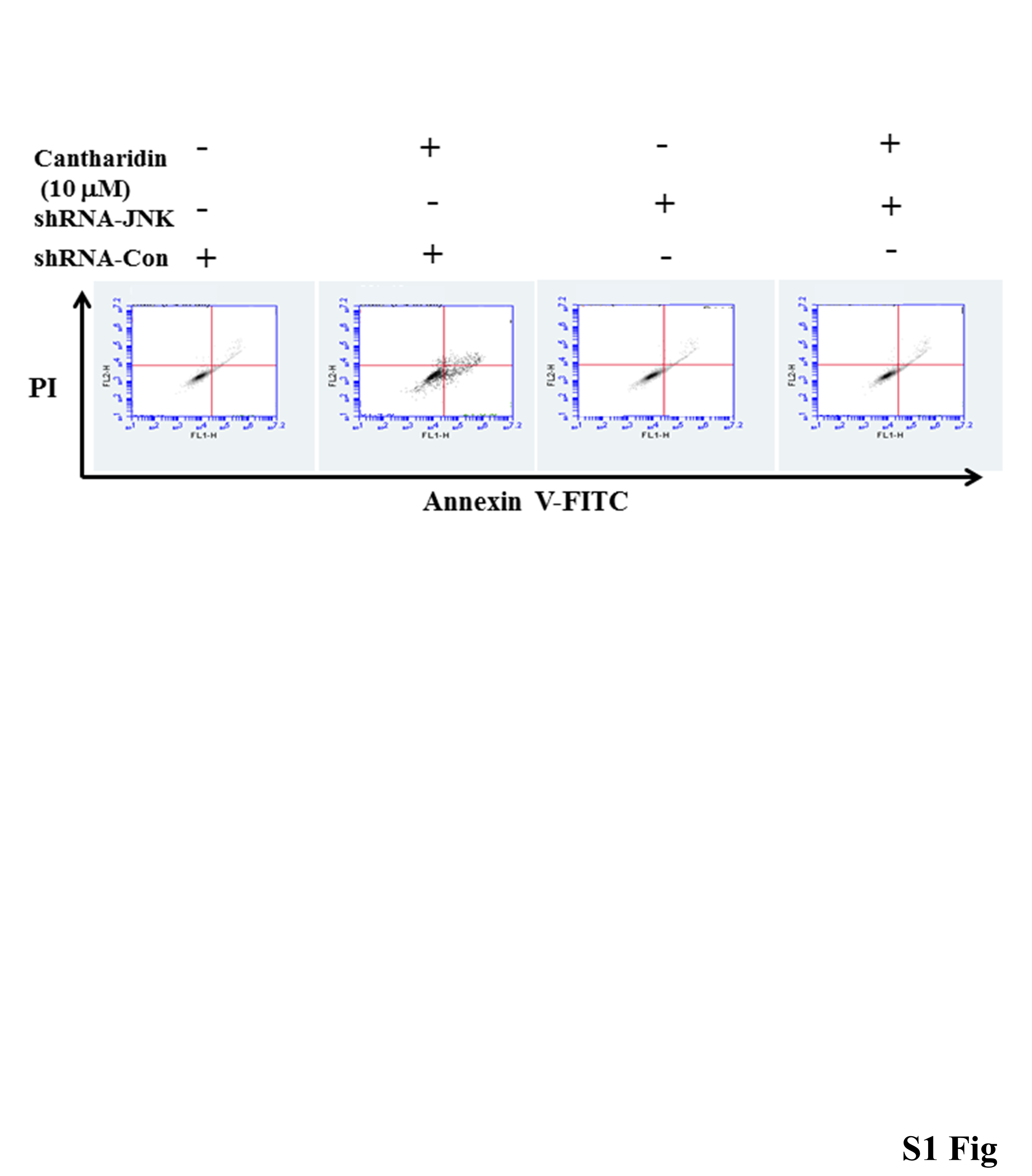

Supplement: S1 Fig — (TIF) [file pone.0168095.s001.tif]

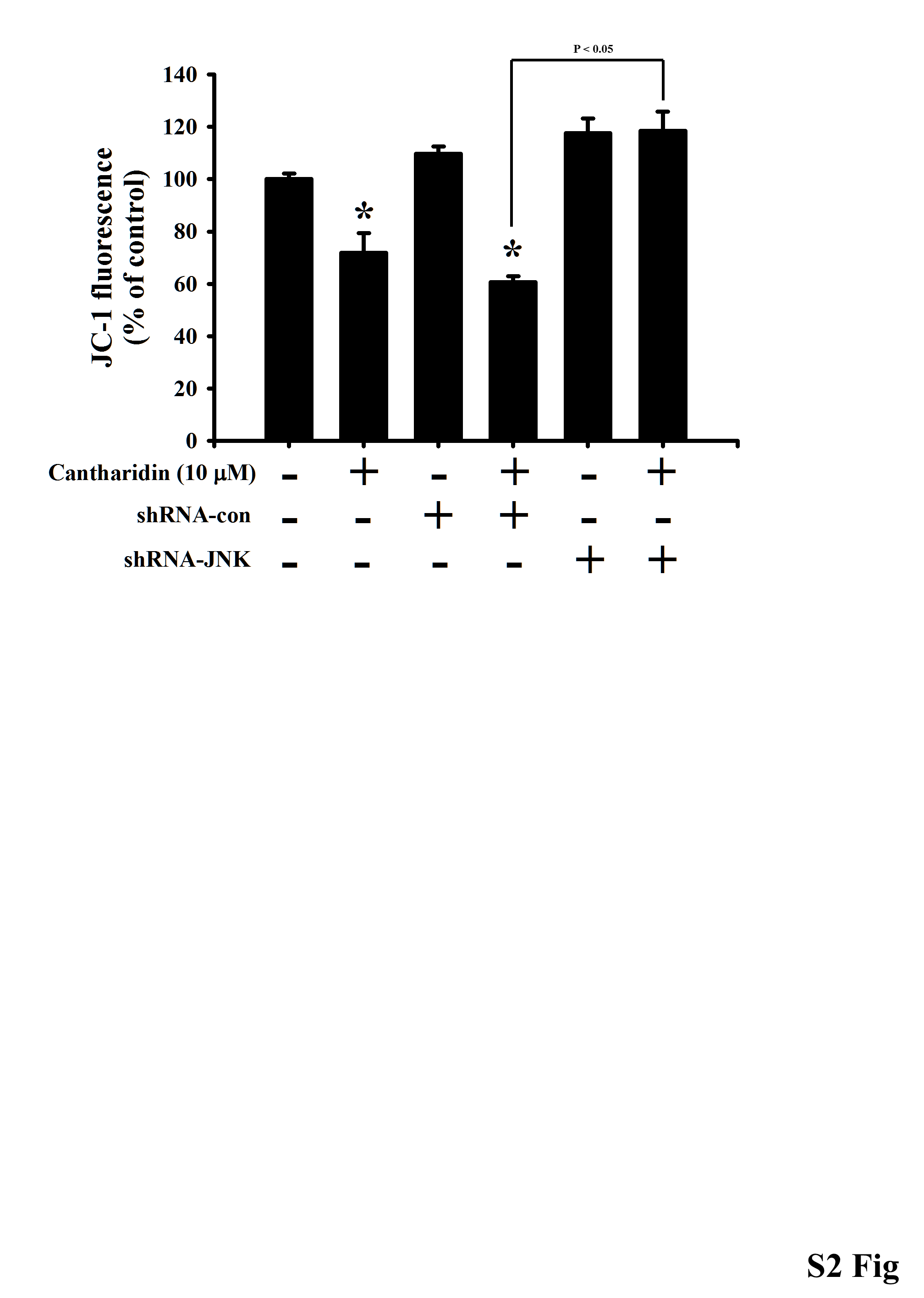

Supplement: S2 Fig — (TIF) [file pone.0168095.s002.tif]
